# A Social Identity Analysis of Climate Change and Environmental Attitudes and Behaviors: Insights and Opportunities

**DOI:** 10.3389/fpsyg.2016.00121

**Published:** 2016-02-11

**Authors:** Kelly S. Fielding, Matthew J. Hornsey

**Affiliations:** ^1^School of Communication and Arts, The University of Queensland, BrisbaneQLD, Australia; ^2^School of Psychology, The University of Queensland, BrisbaneQLD, Australia

**Keywords:** social identity, intergroup, norms, climate change, pro-environmental attitudes, pro-environmental behavior

## Abstract

Environmental challenges are often marked by an intergroup dimension. Political conservatives and progressives are divided on their beliefs about climate change, farmers come into conflict with scientists and environmentalists over water allocation or species protection, and communities oppose big business and mining companies that threaten their local environment. These intergroup tensions are reminders of the powerful influence social contexts and group memberships can have on attitudes, beliefs, and actions relating to climate change and the environment more broadly. In this paper, we use social identity theory to help describe and explain these processes. We review literature showing, how conceiving of oneself in terms of a particular social identity influences our environmental attitudes and behaviors, how relations between groups can impact on environmental outcomes, and how the content of social identities can direct group members to act in more or less pro-environmental ways. We discuss the similarities and differences between the social identity approach to these phenomena and related theories, such as cultural cognition theory, the theory of planned behavior, and value-belief-norm theory. Importantly, we also advance social-identity based strategies to foster more sustainable environmental attitudes and behaviors. Although this theoretical approach can provide important insights and potential solutions, more research is needed to build the empirical base, especially in relation to testing social identity solutions.

## Introduction

The seriousness of environmental issues currently facing the world is increasing despite substantial research attention and the efforts of local, national and international environmental organizations. Climate change is a vivid example of this. Despite being one of the most important environmental challenges of our time, progress on developing effective policy and targets to mitigate climate change has been slow, in part because of the skepticism of segments of society, usually ideological conservatives, who question the reality or anthropogenic basis of climate change. The stark divide between those on the left and right of politics in relation to climate change (and environmental issues more broadly; Dunlap et al., 2001; Dunlap and McCright, 2008) is not the only divide in the environmental domain: farmers and scientists come into conflict over water allocation (Poff et al., 2003), communities oppose expansion of mining, because of threats to the local environment (Urkidi, 2010), and rural landholders oppose environmentalists on the protection or reintroduction of threatened species (Wilson, 1997; Opotow and Brook, 2003).

These examples highlight an intergroup dimension of environmental issues: support or opposition to certain environmental issues can hinge on which group you identify with and groups regularly come into conflict over environmental issues. These intergroup tensions and conflicts are reminders of the powerful influence that social contexts and the groups, we belong to can have on our environmental attitudes, beliefs, and actions. Indeed, our environmental behavior and whether we support a particular environmental action or policy may be determined in large part by our group membership. Our aim in the current article is to draw on the social identity approach (Tajfel and Turner, 1979; Turner et al., 1987; Hogg and Abrams, 1988; Hornsey, 2008) as a way to understand the influence of group membership on environmental attitudes and behavior. We note that there is a growing interest in using social identity theory to analyze environmental problems. For example, Colvin et al. (2015a) have drawn on social identity theory to analyze the drivers of conflict in natural resource management contexts. In the current paper, we draw attention to the group-based dimension of many environmental issues, stimulate research that can address the intergroup context in relation to environmental issues, and provide social identity-based solutions that could address the potentially negative outcomes of intergroup contexts.

## The Social Identity Approach

The social identity approach incorporates two interrelated theories – social identity theory and self-categorization theory — which each seek to explain how individual attitudes, emotions, and behaviors are influenced by the group memberships to which we belong. Each theory has different foci – social identity theory has traditionally focused on intergroup relations, whereas self-categorization theory has traditionally focused on intragroup processes – but they each share the same assumptions and meta-theoretical positions. As a result, it has become common to refer to them in the same breath as the “social identity approach,” and this is the language we use here.

Put simply, the social identity approach posits that our self-concept comprises both personal and social identities; personal identity encompasses idiosyncratic aspects of the self, whereas social identities are derived from the groups to which we belong. Social group memberships can be large-scale social categories (e.g., gender, ethnicity), groups we choose to belong to such as professional groups (e.g., psychologist) or interest-based groups (e.g., environmental groups). When a person categorizes in terms of a particular social identity, the categorization process causes an accentuation of similarities between the self and other ingroup members, and an accentuation of differences between the self and outgroup members. Categorization therefore results in an individual’s attitudes, beliefs, and behavior assimilating to the norms of the salient social group and polarizing away from relevant outgroup norms.

Drawing on social comparison theory (Suls and Wills, 1991), social identity theory also posits that, in order to maintain a positive and clear self-concept, group members are psychologically motivated to see their groups as distinct from other relevant groups, and as more positive than other relevant groups. Consequently, ingroup members favor other ingroup members over outgroup members in evaluations and the distribution of resources (for reviews, see Brown, 2000; Hewstone et al., 2002). For example, we judge ingroup members as more likable, knowledgeable, and trustworthy than outgroup members (Tanis and Postmes, 2005; Foddy et al., 2009). Whether or not this ethnocentrism has clear implications for environmental outcomes depends on the nature of the social context; intergroup relations can be more or less harmonious or conflictual depending on the status relations between groups. If status differences are perceived to be legitimate, conflict is unlikely to arise, whereas status differences between groups that are thought to be illegitimate are likely to give rise to intergroup conflict.

In the remainder of this paper, we draw on the social identity approach as a way to understand human–environment relations. There is a small, but growing body of research that has applied social identity principles to understand climate change and environmental attitudes and behavior. There is also research that is not specifically framed by social identity but nevertheless provides evidence of the influence of social identity concepts on pro-environmental variables. The strength of the social identity approach is that it: (1) articulates how the processes that flow from categorizing oneself in terms of a particular group membership could encourage (or discourage) greater commitment to addressing environmental problems and, (2) acknowledges that relationships between ingroups and outgroups could stymy significant progress in advancing environmental policy. We conclude with a set of social identity-based suggestions for advancing significant and positive environmental policy and behavior and recommendations for future research.

## The Social Identity Approach and Environmental Attitudes and Behavior

### Identity and Assimilation to Ingroup Norms

As outlined above, when social identity becomes salient, similarities amongst ingroup members and differences between ingroup and outgroup members are accentuated. As a result, ingroup members assimilate their attitudes and behaviors to ingroup norms and away from outgroup norms. Perhaps the starkest example of this process can be seen in relation to the impact of political party affiliation on climate change attitudes. McCright and Dunlap (2011) have shown that Democrats in the U.S. have greater belief in and concern for climate change than Republicans. These political alignments are confirmed by other U.S. research (O’Connor et al., 2002; Pew Research Center for the People and the Press, 2006) and extend to other countries. For example in Australia, Labor (a center-left party) and Green party supporters and politicians had greater belief in anthropogenic climate change than (conservative) Liberal/National supporters and politicians (Tranter, 2011; Fielding et al., 2012).

Obviously, the relationship between political affiliation and belief in climate change is likely to be bi-directional. Some people will be drawn to a particular party on the basis of their independently formed attitudes toward climate change, meaning that social identity follows individual attitude formation. But a social identity approach would presume that the other causal path – that affiliation influences attitudes – would be even more pronounced. An example of this pathway was provided by Cohen (2003), who showed that partisan Democrats and Republicans responded to the *very same welfare policies* in entirely different ways depending on which party participants were led to believe initiated the policies: when Democrats thought a policy had emanated from the Republican party, they saw the policy as much less moral and acceptable than when the same policy was attributed to the Democrats (and vice versa). This research shows that a message can be rejected or accepted entirely on the basis of the group allegiance of the messenger (Hornsey and Imani, 2004; Esposo et al., 2013). Political identities provide salient attitudes, beliefs, and norms that describe and prescribe party supporters’ views on these issues. The majority of the evidence for this argument is correlational, but one recent study provides experimental evidence. Unsworth and Fielding (2014) showed that when political identity was made salient, participants who were aligned with the conservative Liberal/National parties in Australia had lower belief in anthropogenic climate change and were less likely to support climate change policies than those whose identity was not made salient.

Further evidence for the influence of ingroup norms comes from norm focus theory which differentiates between injunctive social norms which describe what is approved or desired by group members and descriptive norms which describe what the majority of group members actually do (Cialdini et al., 1990). The independent and interactive effects of these norms has been demonstrated in the environmental domain including in relation to littering (Cialdini et al., 1990), towel reuse in hotels (Goldstein et al., 2008), energy conservation behavior (Nolan et al., 2008; Dwyer et al., 2015), recycling behavior (Fornara et al., 2011; Long et al., 2014; Schwab et al., 2014), eco-friendly consumer behavior (Kim et al., 2012), and intentions to take part in a neighborhood climate protection group (Rees and Bamberg, 2014). On the flipside, when American participants were provided with information that Americans are excessive energy consumers (a negative descriptive norm) they were less concerned about climate change and less supportive of climate change policy than when they learned that China was an excessive energy user or they did not receive information (Jang, 2013). In other words, American participants conformed to the ingroup descriptive norm, albeit a negative one.

Goldstein et al. (2008) have also tested whether norms that came from different identities had differing effects on towel reuse. They found that it was the norms that relate to the most relevant and proximal identity—past guests of the participants’ current hotel room – that had the most influence on towel reuse. Although current guests may not necessarily identify strongly with past guests, they share an identity and the behavior of past guests provides the salient script for how to behave in this particular context. Therefore, this finding fits with the social identity approach that people will be guided by the norms of the most behaviorally relevant ingroup in a specific context.

Other research has shown that when norms are misaligned or in conflict it can weaken effects on behavior. For example, when there is a lack of alignment between what the ingroup approves of and what they actually do in relation to energy conservation, ingroup members’ energy conservation intentions are undermined (Smith et al., 2012). Furthermore, perceiving conflict in the pro-environmental descriptive norms across ingroups can either energize or demotivate intentions to act in pro-environmental ways depending on group members’ pre-existing environmental attitudes (McDonald et al., 2012, 2013).

The social identity approach also acknowledges that the influence of ingroup norms should be stronger for those who are more highly identified with the group (Ellemers et al., 1999). For example, social identification with organic consumers predicts willingness to purchase organic products (Bartels and Reinders, 2010; Bartels and Onwezen, 2014), environmental group membership predicts environmental activism intentions (Fielding et al., 2008a), and identification with environmentalists predicts environmental behavior and environmental activism (Dono et al., 2010). Moreover, more strongly identified group members are more likely to intend to engage in ingroup normative behaviors, such as recycling (Terry et al., 1999; White et al., 2009) and sustainable agricultural practices (Fielding et al., 2008b). In other words, the more highly identified group members are, the more salient will be the norms of the group and the more likely they will be to guide behavior. Recent research examining different dimensions of ingroup identification has also shown that it is the self-investment dimension of identification (i.e., importance of and satisfaction with the group) that influenced adherence to ingroup norms relating to reducing carbon emissions (Masson and Fritsche, 2014).

### The Influence of Intergroup Conflict

As we noted previously, negative and competitive intergroup relations may arise when ingroup members perceive illegitimate status differences between their own group and other relevant outgroups (Branscombe et al., 1999; Ellemers et al., 2002). In addition to climate change, many other environmental issues are marked by just this type of context: the issues are highly contested and often involve the imposition of regulations and decisions by powerful outgroups. Examples include the imposition of environmental regulation on farmers; water allocation decisions that trade-off between water for the environment, agriculture, and the community; or the conflict between groups over fracking of coal seam gas. These latter contexts are often seen as ‘David and Goliath’ situations where mining companies and government agencies are perceived to be powerful agents who propagate unilateral decisions. Although social identity theory does not argue that all intergroup contexts lead to ingroup bias and outgroup derogation, the power differences and perceived illegitimacy that flows from these types of contexts can lead ingroup members to perceive the decisions as unfair and to resist them. A study by Fielding et al. (2008b), for example, showed that when rural landholders perceived more negative relations between rural and urban Australia (with urban Australia the site of government who develop regulation that impacts on rural landholders) they had lower intentions to manage their riparian zones.

When people come into conflict over environmental issues or resources, their social identities come to the fore. People stereotype each other in ingroup-favoring ways (e.g., “we are the defenders of the environment, they are the destroyers”) and outgroup members are denigrated and can be morally excluded from the scope of justice (Opotow and Weiss, 2000; Opotow and Brook, 2003). Research by Opotow and Brook (2003) provides evidence of these processes in the context of environmental protection regulation. When a threatened species act was introduced, ranchers and environmentalists came into conflict. The ranchers viewed the threatened species as insignificant, and characterized non-ranchers as inexperienced, irresponsible, and the cause of the problem. In contrast, ranchers viewed themselves as stewards of the environment who made fair environmental decisions. Despite their espoused environmental stewardship, ranchers were wary of government regulation to protect wildlife, a stance that reinforces environmentalists’ notions that ranchers are anti-environment.

Interestingly, some environmental issues can give rise to emergent group identities that center around strongly held positions [i.e., opinion-based groups (McGarty et al., 2009)]. For example, Bliuc et al. (2015) found that U.S. respondents saw their position as a climate “believer” or “skeptic” as distinct social identities in their own right, and that they perceived each other through a hostile intergroup lens. When political affiliation overlaps with these opinion-based identities, identity faultlines can emerge that make objective appraisals of evidence psychologically implausible. Another example of conflict giving rise to emergent social identities is the conflict that has arisen over fracking of coal seam gas in Australia. Unlikely alliances have emerged between environmental group members, farmers, conservative politicians, and media presenters who oppose fracking, with government agencies and mining companies perceived as the salient outgroup (Hutton, 2012; Colvin et al., 2015b).

There are important consequences of these identities that emerge out of environmental conflict. The alignment of climate change attitudes with political party identity lends an intense and competitive intergroup dynamic to what should be even-handed discussions about science and truth. When framed within an entrenched intergroup context, solutions advanced by one political party are likely to be dismissed by political opponents simply because they emanate from the outgroup. Similarly, in relation to the ‘Lock the gate’ movement, the emergent social identity becomes a short cut for deciding whether someone is friend or foe and whether to attend to or trust information from them. To the extent that information comes from people, who are perceived to be aligned with the outgroup, ingroup members are more likely to dismiss it regardless of its veracity (Abrams et al., 1990; Mackie and Queller, 2000; Esposo et al., 2013). In this way intergroup distinctions become entrenched and the potential to reach compromise or develop viable solutions becomes less likely.

Although intergroup conflict can stymy progress on environmental issues, it should be noted that a degree of intergroup conflict is inevitable when pushing for social change, and that the alternative to conflict is often an unhealthy stasis. The social identity model of collective action (SIMCA; van Zomeren et al., 2008) highlights the fact that collective action is an important precursor for change, and that willingness to engage in collective action is partly driven by identification with social groups. In their meta-analysis of the collective action literature, van Zomeren et al. (2008) showed that stronger social identifications were associated with greater willingness to engage in collective action, and that this was particularly the case when identification was measured with respect to a disadvantaged group or a social movement (so-called “politicized” identities). Other research demonstrates that group identification is positively associated with the belief that the group can be effective in reaching its collective goals (van Zomeren et al., 2010), suggesting a virtuous cycle of identification leading to action leading to positive change leading back to identification.

Of course, reality is more complex than this: whether or not collective action readiness is viewed as positive and legitimate will likely depend on the type of collective action (whether it is violent or non-violent; whether it involves trade-offs with economic goals, etc.). Furthermore, although environmental collective action often involves a range of people from many walks of life, research has shown that people hold negative stereotypes of environmentalists as militant, aggressive, unconventional, and eccentric (Bashir et al., 2013). Bashir et al. (2013) showed that participants had lower pro-environmental intentions when they were exposed to an article promoting environmental sustainability written by a journalist who was a typical environmentalist (i.e., a person who organizes rallies to protest harmful chemicals) than one who was an atypical environmentalist. With this in mind it is easy to see how messages that emanate from environmental groups that are perceived to be extreme may gain little traction with the broader populace and could even polarize people away from support for important environmental issues (Bliuc et al., 2015).

### The Fluidity of Social Identity

The social identity approach recognizes that social identities are not fixed; rather, they are dynamic and flexible, changing in people’s minds as a function of the comparative context. Two key factors influence which social identities guide behavior: fit and accessibility (Oakes et al., 1991). Comparative fit refers to the degree to which a social identity is seen to reflect real world differences between groups. Normative fit recognizes that categorization is a dynamic process that reflects the perceptions of perceivers; that is, people are more likely to categorize into ingroups and outgroups if differences between groups align with stereotypic expectations. Social identities are also more or less likely to become the basis for self-definition depending on how accessible they are; some are fleetingly accessible if primed (e.g., one’s identity as a guest in a hotel room) whereas others are chronically accessible because they are frequently activated (e.g., a workplace social identity).

Some recent research demonstrates the fluid nature of social identities in the environmental domain. Rabinovich et al. (2012) showed that British participants judged the British as more pro-environmental when a less environmental nation (USA) was the salient outgroup comparison whereas the British were judged to be less environmental when a more environmental nation (Sweden) was the salient outgroup. Moreover, participants’ environmental values, intentions and behavioral choices shifted in line with the national stereotype. Thus, the stereotype of the group and group members’ self-conception polarized away from the comparison outgroup. In a similar vein, when students compared themselves to past students (assumed to be less pro-environmental) they judged current students to be more pro-environmental but when comparing current students with future students (assumed to be more pro-environmental) they judged current students to be less pro-environmental (Ferguson et al., 2011). Willingness to engage in sustainable behaviors also varied in line with the perceived ingroup norms, that is, there was greater willingness when participants compared with past students than when they compared with future students.

These findings demonstrate how the intergroup comparative context can influence the content of social identity in ways that could facilitate or inhibit greater engagement in pro-environmental behavior and greater support for pro-environmental policy. Although communicators might intuitively be tempted to highlight a relevant outgroup’s superior environmental record as a way to motivate ingroup action, this may not be fruitful as it could send a negative descriptive norm message to ingroup members. On the other hand, highlighting the superior environmental record of the ingroup relative to other salient outgroups could help to construct a more pro-environmental ingroup stereotype that may have flow-on benefits in terms of influencing ingroup members’ own environmental behavior or their support for environmental policies.

## Integrating the Social Identity Approach with Other Relevant Theories

A question raised by our analysis is whether the social identity approach adds to the understanding of environmental problems beyond other prominent theoretical frameworks. In this section, we examine the similarities and differences between the social identity approach and other relevant theories, with a view to highlighting possibilities for integration and stimulating future directions across frameworks.

One of the most prominent theoretical lenses applied to understanding climate change beliefs is cultural cognition theory, which adapts the theorizing of Douglas and Wildavsky (1982) on cultural influences and risk perceptions. The theory of cultural cognition (Kahan, 2010; Kahan et al., 2010) draws on Douglas’s grid/group taxonomy – *individualistic-communitarian* and *hierarchical-egalitarian –* and argues that these cultural orientations shape people’s appraisal of risk, evidence, and scientific consensus. For example, people who subscribe to relatively individualistic and hierarchical values favor self-reliance, competition and free market solutions. In contrast, people who subscribe to relatively communitarian and egalitarian values are concerned with social injustice, are suspicious of authority (including industry) and are committed to cooperation. Hence, those high on individualism and hierarchy are more inclined to value industry, downplay its risk to the environment and oppose regulation. Research guided by this theoretical framework has shown empirical evidence of the influence of these cultural values. For example, judgments of expertise were influenced by the extent to which that expert’s position aligned with participants’ cultural values in relation to climate change, nuclear waste, and gun laws (Kahan et al., 2011). Specifically, when an expert presented climate change as a high risk, egalitarian communitarian participants were much more likely to agree that they were an expert than hierarchical individualist participants and vice versa, when the expert presented climate change as a low risk (see also Price et al., 2014). Indeed, a meta-analysis shows that ratings of hierarchicalism and individualism share robust and medium-sized relationships with people’s skepticism that anthropogenic climate change is real (Hornsey et al., in press).

What the cultural cognition and social identity approach share is the notion that people filter information through a particular lens—either through the lens of worldviews (in the case of cultural cognition theory) or through the lens of social identity and its associated norms. Hence, both perspectives conclude that beliefs about climate change will depend on how climate change aligns with these important meaning-making psychological structures. Where the social identity approach departs from cultural cognition is in its focus on the context-dependent nature of identity. Worldviews and values are relatively static or at best slow to change whereas identity is fluid and may become more or less salient depending on the context. If we integrate across the two perspectives, cultural cognition theory suggests that people with individualistic and hierarchical values would be more likely to identify with conservative political parties. If they do so and the identity becomes salient, then the norms of that identity will guide responses to issues that are group-relevant, such as climate change policy. This integration suggests that identity may mediate between cultural worldviews and environmentally related attitudes and behavior.

One can also imagine, though, that some contexts may bring to the fore identities that would trump or at least attenuate cultural worldviews. For example, in a workplace where climate change policy is being supported and reinforced, social identity theory would predict that the work-place identity would be the most proximal influence on an individual’s climate-change attitudes and behaviors, at least for those individuals who identify with their organization. For those who are less identified, their worldviews may have a greater influence on their attitudes to the workplace climate change policies. Future research that integrates across these two theories could test these hypotheses.

Within the environmental psychology literature there are two key theories that often frame research seeking to understand environmental decisions and behavior: the theory of planned behavior (TPB; Ajzen, 1991) and value-belief-norm (VBN) theory (Stern et al., 1999; Stern, 2000). The attraction of the TPB is that it is a parsimonious model—it proposes that attitudes, subjective norms, and perceived behavioral control predict intentions which in turn predict behavior. Another advantage of the model is that its simplicity allows other relevant variables to be integrated into the model thereby increasing its predictive power (Conner and Armitage, 1998). Social identity researchers have integrated social identity concepts into the TPB in two main ways: First, they have drawn on social identity theory to address the question of why subjective norms often emerge as the weakest predictor of intentions (Armitage and Conner, 2001). From a social identity perspective, it is not necessarily the norms of important others *in general* that will predict environmental behavior intentions, but rather the norms of the most behaviorally relevant group (Terry and Hogg, 1996; Terry et al., 1999). Terry et al. (1999) showed that perceived norms of a behaviorally relevant reference group were related to recycling intentions for group members who were strongly identified. Fielding et al. (2008b) have also incorporated perceptions of the intergroup context, specifically urban versus rural relations, into the TPB to predict farmers’ intentions to engage in sustainable natural resource management. They showed that these intergroup perceptions emerged as an additional predictor above and beyond the TPB variables. Hence, the social identity approach complements the TPB and can increase its potential to understand and predict environmentally related behavior. It clarifies which norms are likely to influence behavior and highlights the potential of the intergroup context to influence environmental intentions.

Another well-established approach to understanding environmentally significant individual behavior is the VBN theory proposed by Stern et al. (1999) and Stern (2000). This theory proposes a causal sequence that moves from stable values and ecological worldviews to an awareness of consequences for the valued object (e.g., the environment). This in turn influences one’s sense of responsibility to act, which in turn influences personal norms (conceived as an individual’s sense of personal obligation to act on behalf of the environment). At first glance the centrality of personal norms in the VBN runs counter to the primacy of social norms in the social identity perspective. However, the social identity approach conceives of the self as made up of social identity *and* personal identities and so these two approaches are not contradictory. Moreover, the VBN is derived from Schwartz’s Moral-Norm-Activation theory and Schwartz (1973) argued that individual expectations that underpin personal norms stem from shared social norms (see Bratt, 1999 for a demonstration of this relationship in relation to recycling). Stern (2000) also acknowledges that there are a range of factors that feed into environmental behavior, including features of the personal, social, and economic context, and that the influence of personal norms on behavior will depend on the importance of contextual factors. Where the influence of contextual factors are strong, attitudinal factors as outlined in the VBN will be relatively weak predictors of environmental behaviors.

This conceptualization allows a comfortable co-existence between the social identity approach and the VBN—in some circumstances personal norms will be the main motivator of behavior whereas in others group-based social identity considerations will come to the fore. Rather than seeing these two theoretical approaches as parallel processes, though, one could also imagine a feedback loop between social and personal identity. Being members of social groups that value the environment could lead ingroup members to internalize these group norms so that they become a strong personal norm. Of course, it is also possible that individuals join groups on the basis of their values and so having environmentally oriented values predisposes people to joining groups that reinforce those values. Ultimately, longitudinal research is needed to disentangle the causal sequence. Short of this, one might expect that personal norms would be strengthened or weakened depending on the social identity that is salient and whether the norms of that identity align with one’s personal norms.

## Social Identity Strategies to Encourage More Positive Environmental Outcomes

If we accept that the social identity approach offers a helpful theoretical lens through which to examine environmental attitudes and behavior, then it should also be able to offer solutions to address environmental problems and conflicts. We provide some social-identity based strategies below and a summary can be found in **Table 1.** They are not exhaustive but are instead meant to provide a starting point that can stimulate future research to empirically test and further refine social identity approaches relating to the environment. Although some of these strategies have been discussed previously (see e.g., Reynolds et al., 2015; Ferguson et al., in press), we see a benefit in presenting the range of strategies that emerge as the logical outcome of our social identity analysis of environmental attitudes and behavior.

**Table 1:** Social identity strategies to encourage more pro-environmental attitudes and behaviors.

Social identity strategy	Example study
**Use ingroup messengers**	
Ingroup sources are influential because they are perceived to be more trustworthy and credible by ingroup members	Schultz and Fielding (2014). Messages about an alternative water source were more influential when coming from a scientist with a shared regional identity
**Forge a superordinate identity**	
A superordinate social identity can help to reduce intergroup conflict because it subsumes conflicting subgroup identities and transforms the group context from one of ‘us’ and ‘them’ to ‘we’	Samuelson et al. (2003). Conflict over watershed restoration was transformed through forging a superordinate identity in a collaborative learning setting that allowed consensus to emerge and recommendations to be developed
**Link social identity and pro-environmental outcomes**	
Identifying with a pro-environmental group will lead group members to conform to the pro-environmental attitudes and behavior of that group	Van der Werff et al. (2014). Reminding people of their past behavior can strengthen their identification as a pro-environmental person and increase future pro-environmental actions
**Promote pro-environmental ingroup norms**	
Providing messages that highlight the ingroup’s pro-environmental norms will increase group members’ pro-environmental attitudes and behavior.	Nolan et al. (2008). Messages that a majority of householders in the neighborhood saved energy reduced household energy use
Negative descriptive norms can be attenuated by:	
• emphasizing the pro-environmental injunctive norm (i.e., what group members approve of)	
• make salient a superordinate identity that does have pro-environmental descriptive norms	Schultz et al. (2007). An injunctive norm countered the effect of a negative descriptive norm in relation to energy use
• provide a comparison that makes the ingroup appear more pro-environmental	Rabinovich et al. (2012). British participants thought of themselves as more pro-environmental when compared to the U.S.
• leaders can advocate a pro-environmental vision of the ingroup	Seyranian (2014). Group leaders who used inclusive language influenced group members support for renewable energy


### Use Ingroup Messengers

When thinking about how to promote more positive pro-environmental outcomes, an important consideration is where the messages come from. Consistent with social identity theory we know that ingroup sources are perceived to be more trusted and credible and therefore more influential (Hornsey et al., 2002; Kahan et al., 2011). This suggests the need for pro-environmental messages to come from ingroup members whenever possible.

Of course, this may not always be possible; sometimes the environmental issue is a scientific or technical one requiring specific expertise. It may be possible even in this case for the outgroup spokesperson to emphasize a shared superordinate identity with the audience. As an example, Schultz and Fielding (2014) showed that when a scientist provided information about recycled water—a potentially contentious solution to address water shortage situations—and emphasized the social identity she shared with participants (i.e., they all resided in a particular region), highly identified participants had greater support for this sustainable water source.

### Forging a Superordinate Identity to Reduce Intergroup Environmental Conflict

Conflict between groups on environmental issues has the potential to impede progress on addressing these issues. As we outlined above, intergroup conflict reinforces the boundaries between groups so that group members relate more to each other as group members, and are therefore more likely to exhibit ingroup-favoring attitudes and behaviors. When thought of in this way it is easy to see how this type of intergroup context can stand in the way of developing bi-partisan climate change policy, or sustainable resource allocation that benefits the environment as well as other stakeholders.

One way that negative intergroup relations could be transformed is through forging a more inclusive superordinate identity that encompasses conflicting subgroups (Gaertner et al., 1993; Gaertner and Dovidio, 2000; Opotow and Brook, 2003). Focusing sub-group members on a higher order, superordinate group identity helps to shift the context from one of ‘them’ and ‘us’ to ‘we’. Past research has shown that this strategy can reduce prejudice and discrimination (Gaertner and Dovidio, 2000) because outgroup members are now part of the ingroup and are therefore accorded the benefits of ingroup membership. Batalha and Reynolds (2012) highlight the importance of superordinate identity as a way to develop more effective global negotiations around climate change mitigation. Drawing on the Actualizing Social and Personal Identity Resources model (ASPIRe model; Haslam et al., 2003), they argue that negotiations may be more effective if subgroups are formed that reflect the mutual concerns and interests of like-minded nations and that these subgroups then work together to forge a superordinate framework. The ASPIRe model provides a process for forming superordinate identity beginning with identifying the current social identities that people use to define themselves, followed by the formation of subgroups and the articulation of subgroup goals. The final steps involve the overarching organizational group—incorporating all subgroups—formulating superordinate group goals that inform subsequent action. More broadly, the social identity literature suggests that conditions for making a group such as a superordinate identity ‘real’ are accessibility of the identity, fit (as discussed in section “The Fluidity of Social Identity”), and entitativity (i.e., a combination of interdependence, common fate, physical proximity, similarity; Oakes et al., 1991; Sherman et al., 1999).

Samuelson et al. (2003) also provide a concrete example of how conflict between stakeholders over watershed restoration efforts can be transformed through forging a new superordinate identity. The formation of the San Antonio Watershed Council involved bringing a variety of stakeholders together in a structured communication setting that allowed collaborative learning. The formation of the new group identity allowed stakeholders from different subgroups who came with opposing positions to reach consensus and develop a set of recommendations to improve the quality of the watershed.

Forging a superordinate identity, though, should not entail group members losing or negating their subgroup identity. In fact, research has shown that there are greater reductions in intergroup bias when people identify with both their subgroup and a superordinate group simultaneously (Hornsey and Hogg, 2000a,b). In the context of intergroup environmental conflicts, Opotow and Brook (2003) argue that retaining subgroup identities can allow the positive attitudes that can develop because of shared superordinate group identity to generalize to the broader subgroup. For example, in the context of the conflict relating to fracking of coal seam gas in Australia, the Lock the Gate Alliance includes farmers and environmentalists, groups that are usually not in alliance. To the extent that Lock the Gate members retain their subgroup identities, this makes it more likely that farmers and environmentalists can develop more positive, nuanced and less stereotypical impressions of each other’s groups. Preserving subgroup identities also reduces the risk that subgroup identities are threatened (Hornsey and Hogg, 2000a) and allows an appreciation of the distinctiveness of subgroup identities such as the expertise and experiences of specific subgroups (Opotow and Brook, 2003).

### Linking Identity and Pro-environmental Outcomes

From a social identity approach, a simple way to promote more positive pro-environmental outcomes is to make salient an identity that incorporates pro-environmental norms and/or to provide pathways for people to identify more strongly with pro-environmental social identities. A simple demonstration of the latter approach is provided in research by Van der Werff et al. (2013, 2014) although their focus is on making salient self-identity rather than a group identity. They show that reminding people of their past pro-environmental actions leads them to strengthen their identity as a pro-environmental person which subsequently leads to further pro-environmental behaviors. It is easy to envisage how this approach could be scaled up through simple messages that ask people to reflect on their various past actions that help to protect the environment. Campaigns that address local environmental issues, such as drought, are also an opportunity to showcase pro-environmental norms (i.e., relating to water conservation) as a defining element of the identity. Activating the regional identity could thereby make salient and strengthen the water conservation norms and could result in lower ongoing water consumption in the region.

### Promoting Pro-environmental Ingroup Norms

Following on from the point above, there is strong evidence that people are more likely to act in environmentally friendly ways when the norms of a behaviorally relevant ingroup—especially one that people identify highly with—are supportive of pro-environmental action. It may not always be possible to make salient a social identity with supportive environmental norms and, as we noted previously, it is often the case that ingroups have positive environmental injunctive norms but negative environmental descriptive norms (i.e., most group members support pro-environmental action but only a minority actually engage in it). Research has shown that placing a greater emphasis on the injunctive norm can help to overcome the problem of a negative descriptive norm (Schultz et al., 2007; Smith and Louis, 2008). Thus, rather than drawing attention to the negative ingroup descriptive norms (e.g., only a minority of young people are engaging in actions to protect the environment) the focus needs to be on the positive injunctive norm (a majority of young people support actions that protect the environment).

If ingroup norms are not pro-environmental, another strategy supported by the social identity approach (and discussed above) is to make salient a higher order social category that does have pro-environmental norms. For example, data show that young adults engage in less pro-environmental behavior than older age groups (Eurobarometer, 2011). Hence, making a higher order identity salient—for example, a national identity that encompasses more pro-environmental age groups—may help to reinforce pro-environmental norms and positively influence pro-environmental behavior as these norms and behavior can be truthfully attributed to the broader ingroup. This strategy may be more likely to be effective when the lower order category (e.g., young people) are represented as part of the broader social group (Hornsey and Hogg, 2000a). For example, communication that reminds people that members of their country support pro-environmental policy and behavior could be accompanied by images that incorporate a range of citizens including younger citizens. Whether or not this approach could work in contexts where the subordinate identity is particularly salient, such as young people making environmental decisions in the presence of their peers, remains an empirical question.

To this point, we have described strategies that could help to shift perceptions of ingroup norms. A more direct approach is suggested by Seyranian (2014) and Seyranian et al. (2015) who advance the concept of social identity framing as a way to shape the content of ingroup identity. This approach outlines a process whereby a leader can shift social identity content in a direction that can help to promote positive social change. They highlight the need for leaders to advance a vision to group members through the use of inclusive ingroup language (e.g., we, us). As an example, when ingroup leaders advocated for renewable energy using inclusive language, support for renewable energy was perceived to be more ingroup normative and there were greater intentions on the part of ingroup members to act in relation to renewable energy (Seyranian, 2014). Further research is needed to identify the critical elements that are most effective at changing the content of ingroup identity.

## Future Directions

Throughout this paper, we have highlighted interesting and important questions that could be pursued by researchers within a social identity framework. In particular Section “Social Identity Strategies to Encourage More Positive Environmental Outcomes” highlighted social identity-based solutions, many of which need further testing to verify their effectiveness and boundary conditions. In considering directions for future research, we encourage researchers adopting a social identity approach to focus on issues that can have significant environmental impact. Psychological research in the environmental domain has been heavily weighted toward individual actions, such as recycling, or energy and water conservation. But there is a need to expand our focus and to pursue dependent variables that represent greater impact.

In light of the lack of progress in instituting climate change and environmental policy in most countries, an important focus for social identity researchers should be on policy acceptance and providing communicators with the tools to convince people of the need for environmental protection policy. From a social identity approach perspective, lack of policy acceptance reflects that environmental goals are not normative in many groups and this may arise in part because of intergroup conflicts that create divisions rather than bridges between groups. An important challenge for social identity researchers is to identify frames that can appeal to decision-makers and unite disparate groups who conflict over environmental issues. For example, what is the best way to frame climate change policy that will elicit positive responses from political conservatives and liberals alike? Researchers have begun to recognize the importance of finding frames that appeal to differing values and ideologies, for example, Feygina et al. (2010) showed that conservatives were more likely to endorse environmental protection when it was framed as protecting the American way of life and Bain et al. (2012) demonstrated that framing climate change responses as making society a better place or as stimulating development increased climate change skeptics’ environmental citizenship intentions relative to a frame that focused simply on environmental protection. Focusing on frames that appeal to decision-makers and elites may be particularly important given their power to appeal to the broader group. At present there is little research to provide evidence for what works and what does not.

Another important focus for social identity researchers is understanding people’s willingness to take part in collective action to protect the environment. In Section “The Influence of Intergroup Conflict,” we highlighted that the magnitude of environmental problems means that collective action will be needed if we are to effectively address many environmental issues, a point that has been echoed by leading environmental psychology scholars (e.g., Stern, 2000). Effective collective action has the potential to sway segments of the community who do not currently have an opinion or stake in an issue and to send messages to elites and decision-makers. As we noted previously, SIMCA has clearly demonstrated that social identity is a key predictor of collective action and that the more politicized the identity, the stronger the relationship (van Zomeren et al., 2008).

Traditional forms of collective action have centered on taking part in protests or rallies. Marching alongside people who share your beliefs and vision can evoke a sense of shared group identity and potentially reinforce the sense that the group can effectively address environmental problems, thus helping construct a politicized group identity (cf. van Zomeren et al., 2010). But the proliferation of new media and communication technologies is changing the nature of collective action with many groups existing online and with little or no face-to-face interaction amongst members. On the one hand these new ways of conducting collective action can reach large international audiences; online campaigns are sometimes viewed by millions of people and result in swift responses on the part of business and decision-makers. This raises the possibility that this type of collective action may result in a stronger sense of efficacy (than for example more traditional protests), although it also raises the question of what group identity it would foster. The social identity approach seems well-placed to provide a framework for investigating and understanding these new forms of environmental collective action (e.g., McGarty et al., 2014) but work on this is nascent and to our knowledge has yet to be applied to the environmental context.

Some approaches to building support for addressing climate change and environmental problems are coming from grass root movements that bring together people in small groups to build a sense of efficacy to change behavior (e.g., Staats et al., 2004; Dowd et al., 2012). Although these grassroots approaches may vary in format, at their core is the notion that being part of the group will empower people to make changes to their own lives and to potentially become role models for others who are not members of the immediate group. Research framed by the social identity approach, though, has had a tendency to focus on large scale categories and groups (e.g., gender, ethnicity, and political identities). The social identity approach has spent less time examining small group mobilization, and this might help explain why the work on grass roots environmental movements has emerged largely independently of the social identity approach. But the gulf between these literatures is starting to be bridged, in particular, work showing how norms and identities are created and negotiated in part through small-group discussion and communication, and how this in turn embeds action within one’s social identities (Smith et al., 2015). To our knowledge these insights have yet to be specifically applied and tested in the context of environmental mobilization. But consistent with the analysis presented in Section “The Social Identity Approach and Environmental Attitudes and Behavior,” a social identity approach emphasizes the importance of small group activities that develop a sense of shared identity, that foreground positive environmental ingroup norms (both injunctive and descriptive), and that help group members build a broader sense of environmental identity that they can transfer to other group situations, such as family and workplace contexts. The social identity approach therefore provides a depth and breadth of theorizing about group processes that could offer insights to maximize the effectiveness of these groups for changing environmental attitudes and behavior.

## Conclusion

It is clear that social identity is a powerful influence on attitudes, beliefs, and actions relating to climate change and the environment more broadly. As we have outlined above, the evidence for this is that: (1) If we conceive of ourselves in terms of a particular social identity, we are more likely to make pro-environmental decisions and engage in pro-environmental behavior if the norms of the group are pro-environmental; (2) intergroup comparisons can change our conception of the ingroup’s environmental credentials which can in turn influence ingroup members’ pro-environmental attitudes and behavior; and (3) negative intergroup relations can act as a barrier to developing solutions to environmental issues, because intergroup bias leads to distrust of outgroup members and less likelihood of developing consensual solutions. Understanding the influence of social identity on environmental decisions and behavior also suggest strategies to promote a more environmentally sustainable world. These include: (1) using ingroup messengers, (2) forging a superordinate identity to reduce intergroup environmental conflict, (3) linking identity and pro-environmental outcomes, and (4) promoting pro-environmental ingroup norms. Past research has gone some way to providing the empirical evidence to support these claims, however, there is still some way to go in testing these social identity-based solutions as well as providing social identity insights to address environmentally significant problems.

## Author Contributions

Both authors collaborated to research and write this review article. The first draft of paper was written by the KF and MH edited and contributed sections to the paper.

## Conflict of Interest Statement

The authors declare that the research was conducted in the absence of any commercial or financial relationships that could be construed as a potential conflict of interest.
